# A methodology for cross-national comparative focus group research: illustrations from discussions about political protest

**DOI:** 10.1007/s11135-019-00887-5

**Published:** 2019-05-15

**Authors:** Maarten Johannes van Bezouw, Anastasia Garyfallou, Ioana-Elena Oană, Sebastien Rojon

**Affiliations:** 1 Department of Sociology, Vrije Universiteit Amsterdam, de Boelelaan 1105, 1081 HV Amsterdam, The Netherlands; 2 Department of Political and Social Sciences, European University Institute, Via dei Roccettini 9, 50014 San Domenico di Fiesole, Florence Italy

**Keywords:** Qualitative methods, Focus groups, Comparative politics, Protest, Political participation

## Abstract

We propose a methodology for comparative cross-national focus group research and illustrate how this methodology is useful for advancing our understanding of political protest. Focus group research allows researchers to study the collective process of meaning making and formation of intersubjective attitudes. This process has been shown to be relevant for how people discuss politics, and how in turn it could influence participation in politics. However, a systematic methodology for examining the influence of the historical, social, and political context in different countries has not been developed hitherto. In order to allow for comparisons between the formation of attitudes in different countries, we put forward several methodological decisions aimed at achieving standardization in cross-national focus group research design. Group composition, recruitment strategies, and moderation style are the key facets of focus group research that need to be standardized in order to make meaningful cross-national comparisons, but more practical considerations in implementing focus groups cross-nationally are also discussed. We illustrate and critically assess the proposed methodology based on data from an international comparative research project in which 80 focus groups were conducted in nine different countries in Europe and Latin America.

## Introduction

Mass mobilization of citizens has influenced political decision-making in countries worldwide and on a wide range of topics. Recent examples include the Indignados movement in Spain and Greece, anti-corruption protests in Romania and Brazil, and student protests against rising tuition fees and education cuts in the United Kingdom (in 2010) and the Netherlands (in 2019). The amount of street protest has increased tremendously in recent years (Granberg 2013) and concerning a wide array of issues (e.g. Aelst and Walgrave 2001). Over the last few decades, social scientists have developed several theories to understand the dynamics of protest. The availability of the necessary resources (McCarthy and Zald 1977) and the political opportunity structure (Kitschelt 1986) have been stressed in mobilization for protests, and political process models have integrated several of these aspects (McAdam et al. 1996). Social psychological approaches (e.g. Klandermans 1997; Van Zomeren et al. 2008) have explored individual-level motivations for participation in collective action, such as perceptions of efficacy and group identification. Nonetheless, a certain element of surprise seems to be part and parcel of many instances of mass mobilization, especially given the contextual differences between various countries (e.g. Kuran 1991; Kurzman 2004). The majority of empirical research on protest departs from the assumption that people have stable, individually held attitudes about protest and participation in protests. However, the formation of these attitudes is rarely straightforward and people tend to change their opinions based on peer influence, current events, and socio-cultural developments (Fishkin 1991; Hollander 2004). In this paper, we develop a methodology for conducting focus groups cross-nationally and argue that this method not only improves our understanding of how attitudes are negotiated in group processes, but also provides an insight into how these attitudes are shaped by a country’s social or historical trajectory.

Social psychological theories claim that the formation of shared opinions within a group is more predictive of participating in collective action than merely identifying with a group (McGarty et al. 2009). Moreover, Schmitt-Beck and Lup (2013) demonstrate that the more people discuss politics within their social circles, the more they participate in politics. This line of research on political talk and political discussions has focused mostly on why and how often people discuss politics, and how this in turn can influence participation in electoral politics (e.g. Bennett et al. 2000; Eveland et al. 2015; McClurg 2006; Pattie and Johnston 2009). However, *how* citizens talk about protest, and especially the cross-national aspect of political talk has not received much scholarly attention. It is likely that the amount and nature of political discussion varies considerably across different countries. For example, Nir (2012) demonstrates the influence of different electoral systems on political talk and Eveland et al. (2015) show that people from collectivistic and individualistic cultures differ in their willingness to engage in political confrontation.

Conducting focus groups in a cross-national setting means finding a balance between wanting to make the data as comparable as possible across countries but also maintaining the context-specificity which this method provides. In this article we will elaborate on how standardization of the research design can be used to achieve this balance. We draw from data collected in the cross-national research project POLPART,^1^ which conducted 80 focus groups in nine different countries, to show how the research design can influence comparability between countries. Moreover, we will show how the political, social, and historical context influence narratives about protest in several countries.

## Focus groups as a cross-national research methodology

Focus groups have been used in the social sciences since the early 20th century (Merton and Kendall 1946) but have become increasingly popular in the late 1980s and 1990s as a tool for both marketing researchers and scientists (Morgan 1996). Since then, focus group research has provided many insights into how citizens think and talk about politics (e.g. Gamson 1992; Perrin 2009; Walsh 2004). On the topic of political protest however, focus groups have not been used as extensively despite some indications that other qualitative methods provide valuable insights into understanding the dynamics of protest (e.g. Drury 2002; Drury and Reicher 2000; Potter and Reicher 1987).

The core feature of focus groups is that data is collected through the interaction between individuals (Morgan 1996); multiple individuals engage in a dialogue focused on the research theme which is guided by a moderator. The intersubjectivity, or shared meaning, expressed in the focus group constitutes the primary unit of analysis (e.g. Stanley 2016), although several intra-group processes can be studied as well (e.g. Krueger and Casey 2014; Morgan 1996; Stewart and Shamdasani 2014; Wiggins 2016). There is considerable flexibility in how focus groups can be used within different methodological traditions depending on the research question, ranging from observation of natural group discussions to very structured discussions or even adding experimental elements to the design (e.g. Della Porta 2014; Duchesne et al. 2013; Gamson 1992; Myers 1998).

The narratives provided by focus group participants stem from a range of sources, including personal experience, news items, and popular wisdom (Gamson 1992). These narratives are situated in a specific socio-political context (e.g. Lup 2015; Stanley 2016) which is more apparent in focus groups than in survey research. Nonetheless, the vast majority of focus group research has been conducted in a single country (see Perelli-Harris et al. 2014 for an exception). Are there specific historical events that shape current narratives about protest? Are discussions more or less consensual due to diverging cultural norms? Studying focus groups cross-nationally allows for comparison between the sources of the narrative that are developed in focus group discussions.

The core aim of our proposed methodology for cross-national focus group research is to allow for meaningful comparisons between countries. Any country comparison in social science is notoriously hard (e.g. Burkhauser and Lillard 2005) and the context-dependency of focus group discussions (Hollander 2004) adds a layer of difficulty. How do we maintain the richness of the data that focus groups provide without treating these insights as unique instances of political discussion? Our methodology is based around standardization of the design across countries. Standardization of the research design allows for interpreting differences in focus group discussions between countries as stemming from differences in attitudes, the political culture, the social context, and other influences than the research design itself.

In making country comparisons based on survey data, one of the key challenges is to ensure equivalence in what is measured by a certain item in different countries (e.g. Burkhauser and Lillard 2005; Dubrow and Tomescu-Dubrow 2016). However, focus group standardization provides additional challenges: not only do the question items need to be understood the same by participants in different countries but the selection criteria and moderating style also need to be similar across countries in order to produce comparable data.

While the main focus of this paper falls on elaborating which methodological decisions can ensure cross-national comparability between focus groups, we believe the guidelines presented here can also be fruitfully applied in other types of comparative research contexts (e.g. across language groups, across regions within one country).

## POLPART focus groups: comparing nine countries

The main objective of the POLPART project is to investigate the issues that motivate citizens to participate in politics and whether they would engage in institutionalized forms of action—such as voting—or non-institutionalized forms of action—for example street demonstrations (Klandermans 2013). In order to investigate how political action is contingent on a country’s historical trajectory or political culture, the POLPART project conducted focus groups in nine democracies. There are several ways to select cases in designing country comparisons, but typically the decision rests on the balance between similarity versus differences between countries (e.g. Seawright and Gerring 2008). The nine countries in the POLPART project all have a democratic political system in common but differ in whether these are old or new democracies. Moreover, among the new democracies, there was a divide between post-authoritarian and post-communist countries, providing sufficiently different socio-political contexts to compare. The Netherlands, Switzerland, Germany, and the United Kingdom represent ‘old’ or ‘mature’ democracies. Hungary and Romania represent a post-communist context, whereas Argentina, Brazil, and Greece represent new democracies, having changed from authoritarian regimes to representative democracies in recent decades. We opted to organize the focus groups in the capital or other major city in each of the nine countries to ensure comparability in the political opportunities (e.g. Koopmans 1999) for the people who were participating in the focus group discussions. Despite targeting a more specific urban population with this strategy, Goodman et al. (2013) argue that in survey research there is similar bias towards urban rather than rural participants.

## Group composition: who participates in the focus groups?

The unique aspect of focus group data is the interactive nature of the discussions therefore the most important methodological considerations are the *size and composition* of the groups (Gamson 1992; Morgan et al. 1998). Compared to one-on-one interviews, focus groups promote self-disclosure to the extent that social homogeneity among the participants is ensured (Duchesne et al. 2013), making it more likely that the participants feel comfortable revealing information to similar others (Morgan et al. 1998). Too much similarity between the participants however will lead to less cross-cutting discussions (e.g. Mutz 2002) where different points of view are being exchanged. Therefore, a balance must be struck between having differing opinions represented, while maintaining social homogeneity within a focus group. This balancing act is complicated in cross-national focus group research where social homogeneity within groups should be similar between countries.

Achieving social homogeneity is usually done by selecting participants on certain comparable criteria. Usually the more countries involved in the cross-national focus group research, the more observable these criteria have to be to maintain the comparative nature. The reason behind this is related to the availability and quality of data on participant characteristics which can be used in a functionally equivalent manner across countries. Selecting certain age groups for example can be easily managed in different countries, and standardized measures for education level (ISCED; OECD 2007) similarly provides a basic measure of attaining social homogeneity. If the research is conducted in different countries where certain sets or clusters of occupations represent a similar social group, selecting participants based on their occupation can also be a fruitful way of creating comparable and socially homogenous groups (Duchesne et al. 2013). Nevertheless, selecting homogeneity criteria remains research topic dependent, especially as it can often involve a trade-off between standardization on some dimensions deemed relevant with regard to the topic of discussion versus non-standardization on others.

Another important consideration is whether to work with a group of strangers who are invited for the purpose of the research or to work with natural discussion groups—for example people who regularly interact such as a group of friends or co-workers. Using natural discussion groups increases the chance that pre-existing power relations and group dynamics, that are usually hidden from the researcher, influence the content and the nature of the discussions. (e.g. Morgan 1996). Studies using natural discussion groups are valuable in cases where the research topic is related specifically to relations within these pre-formed groups. However, when it comes to using pre-formed groups in cross-national focus group research, researchers should pay particular attention to standardization and to making these groups as functionally equivalent as possible.

Group size is mainly dependent on how complex or contentious the topic of discussion is. Topics that are likely to create a lot of discussions and (emotional) involvement from the participants require fewer participants compared to more neutral topics (Morgan 1996). An additional consideration is whether the goal of the research is to get a wide variety of opinions versus in-depth opinions, with the latter requiring smaller groups that do not impose time constraints. The lower and upper limits in terms of group size in general will not go lower than four or exceed ten (e.g. Duchesne et al. 2013; Krueger and Casey 2014; Morgan 1996). Depending on how the groups are composed, there needs to be a decision on how many focus groups will be conducted in each country. Reaching saturation, the point where collecting more data does not add anything new (Bowen 2008), is normally used as a criterion in qualitative research for the amount of data that needs to be collected. There are rules of thumb, for example using between four and six groups usually will provide saturation although the complexity of the topic and desired depth of opinion have to be considered as well (Morgan 1996). An iterative process of adding more focus groups to reach saturation is less feasible in cross-national comparative research. Based on the research question, the relevant selection criteria (e.g. age, socio-economic status) for the participants should be determined, which in turn will provide the initial division of groups. For the number of groups, “better safe than sorry” provides a good rule of thumb, with additional data being less problematic compared to not reaching saturation.

### The POLPART focus group composition

Given that one of the objectives of the POLPART project was to elicit a range of opinions about political participation, we decided to work with strangers rather than natural discussion groups. With the aim of achieving social homogeneity and political diversity in the POLPART focus groups, we created mutually exclusive categories that could be replicated in all countries. We decided to establish groups based on age and education level. Age is an important variable, not only because placing participants from very different age groups may inhibit conversation, but also because many theories explaining political participation, for example post-materialism (Ingelhart 1977) and biographical availability (McAdam et al. 1996), stress the importance of age. Education, which provides citizens with the necessary civic skills and knowledge to influence politics, similarly provides an important factor in political participation (e.g. Verba et al. 1995:305).

We placed participants in socially homogenous but politically heterogeneous (e.g. Duchesne et al. 2013) groups based on a brief screening questionnaire (see “Appendix 1”). In keeping with the principle of social homogeneity, we formed groups based on similar age ranges and education levels. Within these groups we invited participants that differed in their political attitudes (e.g. left–right self-placement) and past political behavior so as to stimulate an exchange of opinions (Duchesne et al. 2013; Myers 1998). We created four age groups: 18–25, 26–40, 41–60 and 61 + . These four age groups were further divided into low and high education groups (see Table 1) based on the International Standard Classification of Education (ISCED)—low education groups consisted of people who did not obtain a university education (ISCED: levels 1–4). High education groups consisted of people with a graduate or post-graduate degree (ISCED: levels 5–6). Additionally, a group of political activists was included to provide politicized opinions in the groups. This group was not structured according to age and education. Instead, participants were chosen based on whether they reported having participated in a street demonstration, political party, or activist group in the last 12 months. We ensured that activists in both conventional (political party) and unconventional (demonstration) political behaviors were present in the group, with different political ideologies if possible.

**Table 1:** Overview of the POLPART focus groups

18–25 Low education	26–40 Low education	41–60 Low education	61 + Low education	Activists
18–25 High education	26–40 High education	41–60 High education	61 + High education	

Based on the participants’ answers to the screening questionnaire (see “Appendix 1”), we are able to compare the POLPART focus groups on several relevant measures. Participants in Greece and the Netherlands on average reported themselves as more left-wing whereas participants in Romania and Argentina reported themselves as more right-wing (see Table 2). There is considerable variation across countries regarding the percentage of participants that participated in street demonstrations and it is noteworthy that the average in Germany was surprisingly high compared to the other Western-European countries (see Table 2). Together with a relatively high score on political interest, this could indicate an element of self-selection bias. The percentage of people who participated in the last national elections was considerably lower in Greece and Switzerland compared to the other countries. Most importantly, however, is the considerable variation (demonstrated by the relatively large standard errors) in left–right self-placement, collective efficacy, and political interest in each country (see Table 2). Moreover, there was substantial variation on these variables within each group in the different countries as well,^2^ suggesting that we achieved political heterogeneity in the group compositions.

**Table 2:** Political attitudes and past political participation of the focus group participants per country (excluding participants in the activist groups)

Country	Left–right self-placement (0–10)	Collective efficacy (0–10)	Political interest (0–10)	Voted in last national elections	Demonstrated in last 12 months
	M (SD)	M (SD)	M (SD)	(%)	(%)
Argentina (N = 48)	5.40 (1.90)	7.43 (1.96)	7.40 (1.58)	100.0	35.4
Brazil (N = 31)	3.96 (2.92)	8.26 (2.65)	7.20 (2.37)	90.3	40.7
Germany (N = 43)	4.79 (1.88)	7.02 (1.68)	8.00 (1.79)	86.0	37.2
Greece (N = 48)	3.62 (2.18)	6.85 (2.57)	7.67 (2.21)	66.7	40.4
Hungary (N = 51)	5.20 (2.84)	6.63 (2.49)	7.37 (1.83)	88.2	11.8
Romania (N = 49)	5.48 (2.74)	7.23 (3.08)	7.18 (2.46)	73.5	22.4
The Netherlands (N = 45)	3.75 (2.12)	7.23 (1.88)	7.07 (2.08)	84.4	15.6
United Kingdom (N = 52)	4.85 (2.47)	7.58 (1.66)	7.00 (2.12)	90.4	3.8
Switzerland (N = 36)	4.97 (1.99)	7.31 (2.28)	6.15 (2.38)	69.4	12.8

Given that political participation is a rather complex and cognitively demanding topic of conversation (Della Porta 2014; Duchesne et al. 2013), we used a range of four to six participants, with a minimum of four participants per group. In order to avoid no-shows, we over-recruited by inviting between seven to nine persons. In some cases, this led to groups larger than six participants.^3^

## Recruitment: how do you recruit focus group participants?

Recruitment of the participants forms the bridge between the sterile, theoretical decision about the size and composition of the focus groups and implementing these decisions practically. More often than not, recruitment will prove to be a tedious task requiring some degree of flexibility between methodological rigor and pragmatism. In general, potential focus group participants can be reached in many different ways, ranging from convenience sampling through existing networks or groups of people, to highly stratified sampling of individuals with very specific characteristics (e.g. Stewart and Shamdasani 2014). Whereas many practical concerns regarding recruitment such as (self-) selection bias, contacting the participants, scheduling, and sending reminders are important, they are extensively covered in existing literature about focus group methodology (Morgan 1996; Stewart and Shamdasani 2014). Standardization again is the leading principle when applying all of these considerations in cross-national focus group research. For example, if incentives are used to attract participants, the value of this incentive has to be adapted to local standards in the country where the focus group is being conducted.

Similarly to other forms of qualitative sampling, it is important to define the sample universe and sampling strategy before turning to the specific recruitment strategy (e.g. Robinson 2014). A specific question that arises with conducting focus groups in multiple countries is whether different recruitment strategies can result in focus groups that are similar in terms of certain key participant characteristics. Some form of pre-screening is needed, based on the sampling criteria, but also on other characteristics that could influence the discussions. For example, on the topic of political participation it is possible to create social homogeneity by forming groups based on education level, but screening here should include additional relevant characteristics such as political interest, political ideology, or past political behavior. If this longer list of selection criteria is administered in each country in the same way, and decision rules for the selection of the participants on the different criteria are established, the recruitment of the participants can be done online or offline, using recruitment agencies, or advertising the focus group discussions in public places.

### Recruitment strategies in the POLPART Project

Within the POLPART project, we used different strategies to recruit participants, allowing for a direct comparison between these strategies based on the participant characteristics. Participants were selected through recruitment agencies in Argentina, Germany, Hungary, the United Kingdom, and Switzerland. In Brazil, Greece, Romania, and the Netherlands, more diverse recruitment strategies were used such as distributing flyers in public spaces such as libraries, community centers, and supermarkets, as well as posting advertisements on social media sites. In the Netherlands, participants for the younger and lower educated groups were recruited through an agency, as they proved to be more difficult to target with advertisements. Despite the differences in recruitment strategies, we standardized the procedure as much as possible. The focus groups were always advertised as a discussion about ‘social issues’, in order to prevent an overrepresentation of politically interested or politically engaged participants. Additionally, in each country the screening questionnaire was administered to every potential candidate and researchers from the POLPART project were responsible for selecting the participants based on the selection criteria described earlier.

We compared the mean scores on several political attitudes in countries employing a recruitment agency versus countries employing field recruitment strategies (flyers and online advertisements) and tested whether there were any significant differences. Table 3 demonstrates that there are no significant differences in political attitudes between field recruitment and agency recruitment countries, suggesting that the recruitment strategy does not contribute to a self-selection bias. The only exception is that respondents recruited through agencies were more likely to have participated in demonstrations than those recruited in the field but this is most likely due to case of Germany where a large number of participants had been active in street demonstrations (See Table 2). Overall, these findings support the idea that having clear and standardized selection criteria that can be applied throughout different countries within the same design can indeed lead to having comparable focus groups cross-nationally.

**Table 3:** Comparisons between participants recruited by an agency or through field recruitment

Variable	Agency (n = 475)	Field (n = 350)	Test of difference	
	M (SD)	M (SD)	t (DF)	*P* value
Personal efficacy	7.15 (11.42)	6.99 (10.86)	− 0.21 (823)	0.84
Collective efficacy	7.54 (5.32)	8.03 (6.39)	1.16 (823)	0.25
Political Interest	7.40 (2.01)	7.23 (2.20)	− 1.15 (822)	0.25
Political ideology	5.03 (2.45)	4.73 (2.83)	− 1.57 (814)	0.11
Participated in demonstration	2.40 (0.86)	2.03 (0.79)	− 6.32 (823)	< 0.001
Contacted a politician	2.51 (0.81)	2.41 (0.80)	− 1.62 (822)	0.11

## Focus group questions: what do you ask participants and how do you moderate the discussion?

### Type of questions

Focus groups offer a wide range of possibilities when it comes to the types of questions that can be asked to the participants. The questions can range from very specific to very broad, include several follow-up questions or not, they can depend on the type of participants, or even include different scenarios for the participants to discuss (e.g. Duchesne et al. 2013). The two key considerations in cross-national focus group research is that the themes covered by the questions asked in each country are the same, and that the questions will be understood equivalently by the participants in each country. Whereas this latter point is harder to achieve, asking the same questions in each country forces the researchers to think about what to ask in a broad way that still covers the main topic of study. At the same time, cross-national focus group research provides less room for a script of follow-up questions specific to each country.

A range of stimuli can be used to trigger participants to discuss a certain topic or give opinions about, for example, a written scenario, video, picture, or audio recording that is presented to them. Duchesne et al. (2013) for example used a board on which the moderator wrote down comments from the participants to create more debate in the discussions. In another international comparative focus group study, Waddington et al. (2009) used scenarios as the main methodological tool in order to compare the response of police officers in six different countries. Different kinds of stimuli can also be used in combination with questions that are asked to the participants. In cross-national focus group research, a decision needs to be made regarding using exactly the same stimuli in each country, or adapt to the local context in order to convey the same meaning.

### Moderation style

Closely related to the type of questions being asked in a focus group discussion, how the questions are asked is especially relevant for the comparative aspect of cross-national focus group research. A basic distinction in moderation style is between directive and non-directive approaches (e.g. Duchesne et al. 2013). By definition, focus groups take place in the grey area between fully controlled experiments and participant observation in a natural setting (e.g. Myers 1998). This means that the moderator is responsible for the amount of control that is taken in opening and closing topics, turn-taking, and eliciting more in-depth discussion by asking specific follow-up questions (Myers 1998). The more control the moderator takes in any of these, the more directive the moderation style is. Differences in moderation style can create substantial differences in how people will discuss certain topics. More directive approaches can create a ‘teacher–pupil’ dynamic, where participants answer to the moderator rather than discuss with other participants (e.g. Duchesne et al. 2013).

For cross-national focus group research, comparability can be achieved in different ways. When a non-directive approach is used, the influence of the moderator on the discussion is limited. Differences in how participants understand the meaning of certain concepts, or which direction they take in answering the question in this case can be attributed to the focus group participants themselves, rather than to specific follow-up questions from the moderator that steer the participants in a different direction (e.g. Duchesne et al. 2013). However, this non-directive approach needs to be considered in conjunction with the type of question that is asked and the specific instructions that the moderators in each country receive: if the questions are broad and the moderator does not intervene when the discussion diverges to other topics, it might become impossible to compare the discussions between countries. In these cases, the moderators in different countries can be instructed to simply refer the participants back to the question that was asked, whereas follow-up questions should be limited to clarification of opinions rather than substantial follow-up questions.

### The POLPART questions and moderation style

The aim of the questions we asked in the POLPART focus groups was to let the participants discuss at length several key themes related to the main questions of the project: what are the issues that people care about most in society, and which kind of political action would they take to address these issues? We developed five broad questions that were all discussed for about 20 to 30 min each (Table 4; see the “Appendix 2” for the full list of instructions for the moderator for each question). Before that, we had a 5-min round of introductions that served two purposes. On the one hand, it forced every participant to say something early in the discussion, preventing people from remaining silent throughout the discussion. On the other hand, asking them about the first thing that comes to mind when they think about politics prompted the general topic of discussion which was especially helpful for the first two questions where politics is not introduced specifically.

**Table 4:** Overview of POLPART Focus Group Questions

–	Round of introduction: please introduce yourself and tell us what is the first thing or word that comes to mind when you think about politics?
1.	Please discuss collectively, which five issues you believe are the most important for our society?
2.	What can people do about these issues?
Break (10 min)	
3.	Some argue that people in our society are not very active in politics. Why do you think some people do not participate in politics?
4.	Which of the following institutions listen to citizens the most? Which of these institutions can do the most for citizens?
5.	Some people argue that we should move towards a system where people, instead of politicians, make important political decisions. What do you think about this idea?

For the second question—about political strategies—and the fourth question about institutions, different prompts were developed to steer the participants towards discussing political strategies and politically relevant institutions and organizations. More specifically, participants were presented with ten pictures representing both institutionalized (e.g. voting) and non-institutionalized (e.g. street demonstration) forms of political participation, following a 10 to 15 min discussion of potential strategies that could be used to influence politics. In question four, we also presented participants with flashcards representing a range of political, civil-society, and business organizations (see “Appendix 2”). The prompts for some groups substantially altered the discussion, especially when they were not aware of certain political strategies or simply did not think of them during the first part of the discussion. Having the same prompts in each country ensured further standardization in terms of the topics discussed, but also demonstrated which strategies and institutions were salient to participants from each country.

We opted for the same non-directive moderating style in each country to promote interaction between the focus group participants, and standardizing the moderating style this way also improved the cross-national comparability of the discussions. In practice, this meant giving clear instructions to each moderator about the goals of the research and the necessity to avoid follow-up questions aside from clarification prompts. Nevertheless, we observed differences between trained moderators that were hired for the POLPART focus groups—for example in Hungary and the Netherlands—and researchers from within the POLPART research team who moderated the focus groups in their country—for example in the United Kingdom. It became apparent that the professional moderators sometimes used more directive techniques to involve different people in the conversation, which created more ‘teacher–pupil’ interactions where participants responded to the moderator rather than other participants.

## Practicalities: what do you need to arrange?

The decisions about who participates in focus groups and how to recruit these people are crucial in a comparative cross-national focus group project. Nevertheless, more practical concerns in the implementation of this design can have a substantial effect on the quality and standardization of the focus groups. Stewart and Shamdasani (2014) provide an excellent overview of the practical considerations regarding arrangement of the groups and recording the discussions. For cross-national focus group research however, every practical decision should stem from a two-step decision making process: how does it follow from the research question or goal of the research, and can this be implemented in the same way in each country?

### Recordings

Recording of the focus group discussions is crucial when verbatim transcripts are needed for the analysis. Although video cameras are generally more invasive compared to audio recorders, they are often times necessary for the transcription of the discussions if not simply to serve as a backup of the audio recording. When the group exceeds four participants, it becomes harder to distinguish who is talking without a video recording. In addition, video recordings allow for interpretations of non-verbal communication as well, if this is part of the research goals (see Onwuegbuzie et al. 2009 for analyzing different types of focus group data). A practical recommendation is the use of small-sized action cameras, that are both less invasive than larger cameras and also capture more of participant interaction because of the wide-angle lens.

### Location and time

Location is an important consideration for maintaining comparability in the research design. Depending on the topic, conducting focus groups in a rural area in one country while choosing the capital of another country for other focus groups can influence the discussions through the availability of similar participants. On the topic of political participation, for example, living in the capital of each country roughly provides the inhabitants with equal opportunities to become politically active (e.g. Koopmans 1999; Kriesi, Koopmans, Duyvendak and Giugni 1992). Similarly, conducting focus groups exclusively during the day in one country and in the evening in other countries weakens the standardization across countries due to possible differences in availability of people working a daytime job for example.

## Cross-national differences in discussions about protest

The second aim of this article is to illustrate how the proposed methodology for using focus groups in a cross-national comparative way can be useful for studying protest. We argued that focus groups provide an insight into the process of intersubjective meaning-making of attitudes (Stanley 2016), adding to quantitative measures of motivations to join political protest. Moreover, in line with Lup (2015), we argued that cross-national focus group research allows for an examination of cultural differences in how these attitudes are negotiated. We will illustrate both of these points with excerpts about street protest taken from the POLPART focus group discussions in different countries, where participants discuss the same question in each country about what citizens can do about important issues in society (see “Appendix 2”, question 2). All the names that are used below, are fictitious names and “M” is used to indicate the moderator.

### Formation of attitudes towards political protest

Observing the intragroup process of attitude formation in focus group discussions shows the value of studying the interaction between participants compared to assuming attitudes are stable factors, as shown in the following exchange in a Dutch focus group:

18–25 LE (the Netherlands)

Linda: I can stand there alone in a field and trying to get people to join [a demonstration], with a time, a date, etc. etc., it costs money and time is what I am saying
Jack: Yes, but if you think something is really important, you take that time. But apparently nobody thinks it is important enough…
Linda: Yes
Jack: … to still do it
Linda: No, but I have the idea that it won’t influence anything. Deep down
Jack: It wouldn’t?
Linda: No. The only thing I do is voting, and even that I haven’t done the last two years I think
M: I basically hear two things now. I am hearing, well I have no time and the feeling that it won’t have any influence. Those seem like two different things
Linda: It depends on both, if you think you will have some influence, you would make time for it
Ciska: Yes
Linda: And if I hear about a demonstration, and I agree with the demonstration, I would make time to actually go there. But really to set it all up…

Linda is seemingly skeptical about the idea of using street protest to influence politics at first, citing practical reasons why it would be hard to organize a protest. Responding to her however, Jack tries to nuance this opinion by saying it depends on the type of issue at hand. Following these initial remarks, the moderator summarizes the different reasons that are mentioned. In the interaction, Linda puts forward several boundary conditions that in the end would make her participate in protest, while making the distinction, that was not there before, between organizing a protest and joining a protest. The information about these boundary conditions and development in attitudes can be derived directly from focus group discussions, whereas measuring attitudes towards protest with survey items entails the risk of assuming stability in attitudes that is not there, or missing different processes that might lead to similar scores on an item. The following exchange between focus group participants in Romania also illustrates how nuances in attitudes that might get lost in survey research can be captured in focus group discussions.

Activists (Romania)

Geo: I can say related to this that at least in the last period I have a bit of doubts, a type of skepticism about this protesting mode of changing something. I mean, I don’t know, maybe I’m becoming conservative lately [people laugh], but it seems to me that this is not the way to change things necessarily, it is more of a thing of the moment. We take down x and put y instead, you know?
Dina: Since’89 this is how people think important decisions can be made
Geo: Yes, and all, at least this thing of the generation, it seems to me that young people are protesting just to protest because, I don’t know, it is the enthusiasm and the faith that
Bia: But let’s not generalize
Geo: No, no, there are situations when protest is really needed and the ones that are there
Bia: … and people that want, yes, yes
Geo: And people that do it for the right reasons, but beyond this I don’t always have faith. I mean this script that young people always do good and that protests are always good.”

Bia explicitly states that not all young people are the same when it comes to protest, shaping especially the way Geo expresses her attitude towards protest in a more nuanced way. It becomes clear here as well that the data stemming from focus group discussions is manifested in between personally held attitudes and collective attitudes (Stanley 2016). Sometimes this means that opinions and attitudes are shaped through different initial points of view like the two exchanges above, but sometimes there will be quick convergence of opinions on a certain topic.

### The role of historical, social, and political context

Whereas intersubjectivity and collective meaning-making are processes in all focus group research, our proposed cross-national focus group methodology allows for studying how social, political, and historical contexts influence focus group discussions. The role of context in shaping attitudes is evident in the examples that citizens provide to defend their views. This point becomes apparent when participants in the POLPART focus groups actively tried to think of instances of protest in their countries in order to form opinions about protest in general. In the United Kingdom for example, participants’ assessments of the efficacy of protest were largely based on their views towards the Poll Tax riots (e.g. Stott and Drury 2000).

26–40 LE (United Kingdom)

Daniel: I think we live in a fake democracy, we think we live in, we are democratic, but we’re not. I think you can protest as much as you like and it won’t make a lot of difference
Several: Yeah
Simon: The last protest that actually worked that I can remember was um under Margaret Thatcher when she done the um poll tax
Julie: Yeah
Simon: They had millions of people in the street and that actually did get scrapped [unclear] -
Julie: But it didn’t make a difference
Simon: But that’s the last one I can remember, that’s like how many years is that? Since then, I can’t remember a single protest that made a huge difference or any considerable difference

Discussing whether protest is effective, the participants in this exchange are searching for specific instances on which to base their arguments. It is clearly an interpretation of the historical and current context that signals a lack of belief that anything can be changed through protesting, even though the Poll Tax riots are mentioned as an example of a protest which achieved its objective, these protests took place many years ago. Similarly, in Greece, people give the example of one wave of protests that they deemed successful to some extent, namely the 2011 Indignant Movement. Participants suggested that, contrasting with previous protests, the Indignant Movement made a political difference because: 1) it encompassed the interests of the population at large, putting aside particularistic interests; 2) staged protests lasting over two months; and 3) provided experimental space for new collective action repertoires such as camps, self-organized solidarity networks, participatory, deliberative and direct democratic practices.

41–60 LE (Greece)

Giannis: Out of all the demonstrations I remember the only massive demonstration that scared the politicians was the situation with the Indignant Movement. That time everyone who looked at the politicians would understand that they were all scared, all the political spectrum
Evaggelia: They [Indignant Movement] were persistent and had duration
Giannis: Because they [politicians] realized there was a very large group of people that were not represented by them
Evaggelia: Exactly
Giannis: Don’t you believe the Indignant Movement changed the situation?
Lina: No
Olga: We didn’t see it
Giannis: Centrist big parties disappeared, isn’t this a change in the political landscape?
Olga: Yes, of course
Giannis: Of course, of course! In my view this was an opportunity for new things to emerge
Eleni: And for other parties to join the parliament.”

Whereas Lina argues that the Indignant Movement was unsuccessful in preventing politicians from voting for austerity, the rest of the participants mobilize to persuade her otherwise. According to them, the Indignant Movement not only made apparent the crisis of representation in Greek politics, but appears to have restructured the party system. Mainstream parties (PASOK and New Democracy) that ruled Greece for 35 years were punished and new challengers were rewarded mainly on the left (SYRIZA).

From both the British and the Greek focus group excerpts, it is apparent that the political and historical context is not the only driving factor in how attitudes are shaped. Rather, it is an interpretation of this context and therefore a reciprocal process between personally held beliefs and the historical, social, and political context that shapes the peoples’ attitudes. In the Dutch focus groups, participants often struggled to think of recent examples of protests simply because there have not been many large-scale protests in recent years. With the lack of saliency of recent protest in the media, participants often expressed some nostalgia about an idealized description of how politically active Dutch people used to be a few decades ago. Taken together, these excerpts from the POLPART focus group discussions illustrate that a better understanding of both attitudes towards protest and the formation of attitudes in focus groups more generally can be more efficiently captured using cross-national comparisons.

## Concluding Remarks

In a political arena where protest is becoming an increasingly conventional strategy to influence politics on a wide variety of topics, focus group discussions provide us with a better understanding of how citizens’ attitudes towards protest are being negotiated and shaped in social interactions, which in turn could influence their propensity to participate in protests. We proposed a methodology for cross-national focus group research, serving two related goals. First, focus groups have seldom been used cross-nationally and we argue that especially due to the contextual influences on how people form attitudes (Hollander 2004; Stanley 2016), using focus groups cross-nationally enhances our understanding of meaning-making and attitude formation across cultures. Secondly, we illustrated the methodology we put forward with findings from a cross-national research project, in which 80 focus group discussions were conducted in nine different countries in Europe and Latin America. We used excerpts from the focus group discussions to demonstrate that attitudes towards protest are not always stable (Fishkin 1991), but sometimes shaped by social interactions and the country’s political culture and historical trajectory.

We see our proposal for a cross-national focus group methodology as a starting point for increased scholarly efforts in this direction and we believe that the principles we outlined can be applied more broadly in comparative focus group research. Despite the focus on attitudes towards political protest, research questions on many other topics can be answered using a cross-national methodology that allows for a meaningful comparison on complex topics of discussion. Similarly, although we focused on the comparability of the focus group participants across groups and countries, we believe the guidelines introduced here are also of interest for researchers seeking comparisons in other domains, for example across language groups, ethnicities and regions. Standardization of the research design can in these instances warrant a meaningful comparison between the group discussions without losing the depth of understanding of social phenomena that focus groups offer.

Future developments of cross-national focus group research could benefit from considering the transformative effect that engagement in focus groups can have on its participants. How much does participation in these collective group discussions alter the participants attitudes, beliefs, or norms? Do people change their behavior after engaging in critical discussions with their peers? We think that using focus group research has tremendous potential in capturing these processes that are relevant for different disciplines in social science. In a time where comparative and, in particular, cross-national research is becoming more and more important, we hope that our proposed methodology provides a first step in applying focus groups comparatively across diverse contexts more regularly.

## Footnotes

1 www.polpart.org.

2 We found *SD *< 1 for 1 out of 80 groups on political ideology, 4 out of 80 groups for efficacy, and 7 out of 80 groups for interest, indicating that nearly all groups were heterogeneous considering the political attitudes of the participants.

3 Additionally, Brazil did not conduct one focus group (61 + LE) due to recruitment difficulties. One group in Brazil had 3 participants, all other focus groups in all countries had at least 4 participants.
